# Outbreaks of autochthonous Dengue in Lazio region, Italy, August to September 2023: preliminary investigation

**DOI:** 10.2807/1560-7917.ES.2023.28.44.2300552

**Published:** 2023-11-02

**Authors:** Gabriella De Carli, Fabrizio Carletti, Martina Spaziante, Cesare Ernesto Maria Gruber, Martina Rueca, Pietro Giorgio Spezia, Valentina Vantaggio, Alessandra Barca, Claudio De Liberato, Federico Romiti, Maria Teresa Scicluna, Stefania Vaglio, Mariano Feccia, Enrico Di Rosa, Francesco Paolo Gianzi, Cristina Giambi, Paola Scognamiglio, Emanuele Nicastri, Enrico Girardi, Fabrizio Maggi, Francesco Vairo, the Lazio Dengue Outbreak Group, Lazio dengue Outbreak Group, Michele Di Donato, Gianmaria Baldin, Francesca Giovannenze, Francesca Raffaelli, Lucia Salvatore, Luisa Sestito, Eleonora Taddei, Antonella Cingolani, Massimo Fantoni, Serena Vita, Samir Al Moghazi, Evangelo Boumis, Angela Corpolongo, Alessia Rianda, Ermenegildo Furino, Cristiana Gianni, Cinzia Barletta, Alessio Pendenza, Giorgio Esterini, Flavio Mellace, Fabio Vivaldi, Adele Gentile, Saul L. Torchia, Federica Mormone, Domenico Barbato, Franca Mangiagli, Roberto Giammattei, Alessandro Zerbetto, Amilcare Ruta, Giorgio Nicolò Malatesta, Gilda Tonziello, Maria Concetta Fusco, Alessandro Agresta, Claudia De Santis, Maurizio D’Amato, Giovanni Pitti, Giulia Matusali, Francesca Colavita, Eleonora Lalle, Licia Bordi, Silvia Meschi, Daniele Lapa, Anna Rosa Garbuglia, Davide Mariotti

**Affiliations:** 1 Regional Service for Surveillance and Control of Infectious Diseases (SERESMI)-Lazio Region, National Institute for Infectious Diseases “Lazzaro Spallanzani” IRCCS, Rome, Italy; 2 Laboratory of Virology, National Institute for Infectious Diseases “Lazzaro Spallanzani” IRCCS, Rome, Italy; 3 Directorate for Health and Social Policy, Lazio Region, Rome, Italy; 4 UOC Diagnostica generale, Istituto Zooprofilattico Sperimentale del Lazio e della Toscana “M. Aleandri”, Rome, Italy; 5 UOC Virologia, Istituto Zooprofilattico Sperimentale del Lazio e della Toscana “M. Aleandri”, Rome, Italy; 6 Lazio Regional Blood Center, Italy; and Department of Molecular Medicine, Sapienza University, Rome, Italy; 7 Lazio Regional Transplant Center, Azienda Ospedaliera San Camillo Forlanini, Rome, Italy; 8 Department of Prevention, Local Health Authority Roma 1, Rome, Italy; 9 Department of Prevention, Local Health Authority Roma 2, Rome, Italy; 10 Department of Prevention, Local Health Authority Latina, Latina, Italy; 11 Highly Infectious Diseases Isolation Unit, Clinical Department, National Institute for Infectious Diseases “Lazzaro Spallanzani” IRCCS, Rome, Italy; 12 Scientific Direction, National Institute for Infectious Diseases “Lazzaro Spallanzani” IRCCS, Rome, Italy; 13 The members of the group are listed under Collaborators; * These authors contributed equally to this work and share first authorship.

**Keywords:** Dengue, Italy, outbreak, autochthonous, vector control, phylogenetic analysis, one-health approach, aedes albopictus

## Abstract

Between August and September 2023, three distinct autochthonous dengue virus transmission events occurred in Lazio, Italy, with the main event in Rome. The events involved three different dengue serotypes. No link with previous imported cases was identified. Here we describe the epidemiological and phylogenetic analysis of the first autochthonous cases and the implemented control actions. The multiple transmission events call for a strengthening of the vector control strategies and future research to better characterise the risk in countries like Italy.

In the European Union and European Economic Area (EU/EEA), dengue viruses are almost always imported from endemic countries. Autochthonous cases are sporadic, and only small outbreaks have been described [1]. In Italy, there was only one outbreak in 2020, linked to a case imported from Indonesia [2]. In 2023, by October 16, 14 Italian regions had reported 215 imported cases and two (Lombardy and Lazio) had reported 58 autochthonous cases [3]. 

Here we present the first autochthonous cases of dengue occurring in Central Italy (Lazio region). The cases were concomitant but linked to three different transmission events.

## Case description

On 18 August, a case (Case 1) was notified to the Regional Service for Surveillance and Control of Infectious Diseases (SERESMI)-Lazio Region with a history of fever followed by maculopapular rash and positivity for dengue IgM and IgG. A serum sample sent to the Regional Reference Laboratory (RRL) of the National Institute for Infectious Disease (INMI) Lazzaro Spallanzani resulted positive for dengue virus serotype 1 (DENV-1) by RT-PCR. Travel, contact with travellers and other possible exposures were excluded, and the case was considered autochthonous.

The Regional Health Authority released an alert note on 22 August to all healthcare facilities, emergency departments and general practitioners in Lazio, recommending considering the diagnosis of dengue even in the absence of a history of travel to endemic countries in patients with acute febrile syndrome and rash, arthralgias or myalgias. Biological samples from suspected cases should be forwarded to the RRL for immediate diagnosis with rapid testing and further serological and molecular analysis.

On 31 August, two persons (Cases 2 and 3) with no travel history were notified, being positive in the DENV-NS1 antigen rapid test; RT-PCR detected DENV-3.

Between 5 and 12 September, three additional symptomatic persons with no travel history (Case 4, 5 and 6) were notified and molecularly diagnosed with DENV-1. 

On 20 September, a symptomatic person with no travel history tested positive for IgM at the RRL; RT-PCR detected DENV-2 (Case 7). A family member, returning from an endemic country and symptomatic, had tested positive for IgG alone on 31 August; upon retesting of the original sample, NS1 antigen was detected, and RT-PCR identified DENV-2.

Median age of these seven cases was 48 years (IQR: 45-66), six were male, one female. All cases were symptomatic. The most common symptoms were fever and myalgia (n = 6), arthralgia and rash (n = 5). Epidemiological and laboratory characteristics are shown in the Table. Date of symptom onset ranged between 2 August and 12 September and cases were notified between one and 16 days after symptom onset. 

**Table:** Epidemiological and laboratory characteristics of the three autochthonous dengue transmission events in the Lazio Region, Italy, 2023 (n = 7)

Epidemiological and laboratory parameters	DENV-1 cluster^a^				DENV-3 cluster		DENV-2
	Case 1	Case 4	Case 5	Case 6	Case 2	Case 3	Case 7
Date of notification	18 Aug	5 Sep	8 Sep	12 Sep	31 Aug	31 Aug	20 Sep
Epidemiological link with imported case	No	No	No	No	No	No	Yes
Laboratory results							
DENV NS1 antigen	NA	Negative	Positive	Positive	Positive	Positive	Positive
DENV IgG IC	Positive	Borderline	Positive	Negative	Negative	Negative	NA
DENV IgM IC	Positive	Borderline	Positive	Positive	Negative	Negative	NA
DENV IgG IF	Positive	Positive	Positive	NA	Weak reactivity	Weak reactivity	Positive
DENV IgM IF	Positive	Positive	Positive	NA	Weak reactivity	Weak reactivity	Positive
DENV RT-PCR (Cq at diagnosis)	31	32	23	27	22	24	36
DENV serotype	1	1	1	1	3	3	2

Cq: quantification cycle; DENV: dengue virus; IC: immunochromatography; IF: immunofluorescence; NA: not available.

^a^ Only the first four cases in this cluster for whom phylogenetic analysis was completed are shown.

Twenty-five additional cases with no travel history have been reported in Rome and province of Rome and linked to the DENV-1 cluster as at 16 October.

## Epidemiological and laboratory investigations

During the epidemiological investigation, Case 1, who lives in the metropolitan area of Rome, did not report travel abroad, blood transfusions, nor occupational or sexual exposures. Their relatives, friends, colleagues and neighbours were asymptomatic and did not report travel abroad. Cases 4, 5 and 6 and the additional DENV-1 cases lived or worked in close proximity to Case 1’s residence. Cases 2 and 3 visited the province of Latina (60 km from Rome) during the 15 days before symptoms’ onset. An imported case lived with Case 7.

To identify possible primary cases, we performed a retrospective evaluation of all 20 imported dengue cases reported in Lazio since January 2023: eight had DENV-2, 10 had DENV-3 and two had DENV-1 infection. A symptomatic DENV-3 case imported from eastern Africa who resided for 2 days in mid-August in the same neighbourhood as Cases 2 and 3 was identified as a possible primary case (Case I-1 in the Figure). No plausible correlation with previous imported DENV-1 cases was found.

**Figure:**
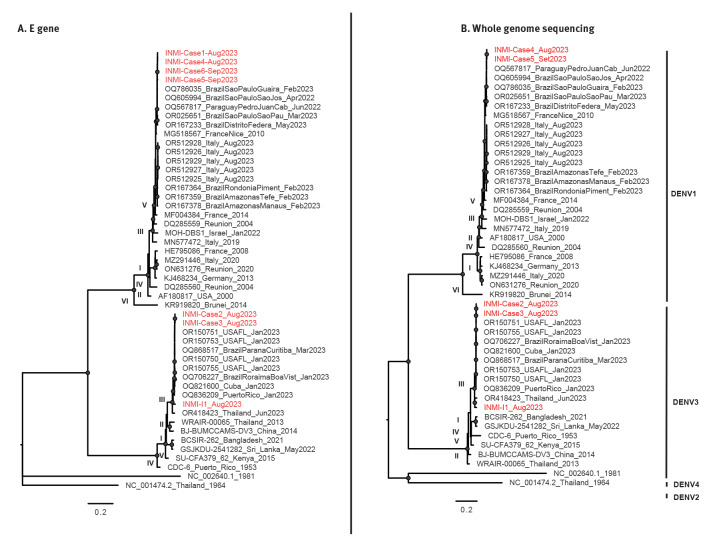
Maximum likelihood phylogenetic trees of dengue virus outbreak isolates, Italy, at 19 September (n = 6) and dengue virus isolate of imported case (INMI-I1)

DENV: dengue virus; INMI: National Institute for Infectious Disease.

Both trees were built using IQ-Tree and ModelFinder to select the best tree model. Bootstrap values were generated using 1,000 replicates. Nodes with more than 80% are reported in bold. The scale bar shows the number of substitutions per site. INMI sequences are highlighted in red colour.

Viral sequencing was performed by Sanger method on samples collected from Cases 1, 4, 5 and 6, using highly sensitive home-made nested PCR with overlapping fragments covering the entire E gene of the viral genome (1,485 nt). Moreover, samples collected from Cases 2 and 3 and Case I-1 underwent whole genome sequencing (WGS) by an amplicon-based approach on the Ion Gene Studio S5 Prime system (LifeTechnologies, Carlsbad, United States) using primer sets designed by Su et al. [4].

Phylogenetic analyses were carried out using INMI sequences (E gene and WGS), and the most recent DENV genomes available in GenBank, including sequences belonging to recent cases in northern Italy and cases reported in Europe (Figure). The phylogenetic tree did not show any direct relationship between autochthonous DENV-3 cases and Case I-1. Phylogenetic analysis of remaining cases is ongoing.

## Vector control activities and public health measures

Intensified mosquito control activities were conducted in a 200 m buffer zone around houses, working places, and other sites attended by the cases for at least 5 h per day, as indicated by the Italian National Plan against arboviral infections [5]. Control was mostly directed at Asian tiger mosquito, *Aedes albopictus*, the only known competent vector for dengue virus present in central Italy and particularly abundant in Rome metropolitan area. Disinfestations included the use of larvicides, adulticides and, when possible, the removal of breeding sites. An entomological monitoring system was set up using BG-sentinel traps, ovitraps and manual aspiration to control the effectiveness of the disinfestations, define mosquito density and evaluate virus presence in the vector. A pool of *Ae. albopictus* was found positive for DENV-1 at the working site of Case 4, confirming the role of this species in allowing viral circulation in Rome.

On 21 August, dengue screening by molecular testing was activated in organs, tissues, haematopoietic stem cells and blood donors who resided in Rome or stayed in Rome for at least one night. The screening was extended to the province of Latina on 1 September, covering the 28 days before donation.

## Discussion

By 16 October 2023, according to the European Centre for Disease Prevention and Control (ECDC), 35 autochthonous cases had been reported in France, and a concomitant DENV-1 outbreak with 30 cases is ongoing in Italy (Lombardy region) [1,3,6]. Before 2023, only one autochthonous dengue outbreak had been reported in Italy; it occurred in northern Italy (Veneto) in 2020, with 10 people linked to a primary DENV-1 case returning from Indonesia [2,7].

Here we have described three distinct dengue transmission events that occurred simultaneously in Lazio; the first involved two DENV-3-infected cases; the second was sustained by DENV-1, involving 29 cases as at 16 October, and is still ongoing. A third event, with one case of DENV-2, suggests intrafamilial transmission; no additional cases were identified.

Although a potential primary case for the DENV-3 outbreak was identified, phylogenetic analysis did not provide a clear epidemiological link for any of the cases. Multiple autochthonous cases, unrelated to identified imported cases and belonging to different serotypes, suggest substantial ongoing viral circulation. In this scenario, with outbreaks occurring in a large metropolitan area, extensive vector control measures become mandatory, as well as all measures to reduce human–vector contact.

Increased human mobility, trade, population growth and climate change constitute risk factors for geographical expansion of vector-borne diseases into new areas [8-11]. As *Ae. albopictus* distribution is expanding globally, more extended areas in the mainland EU/EEA region are at risk for transmission of *Aedes*-borne viruses. With environmental conditions favourable for vector activity and virus replication in vectors, the risk of dengue as well as other arboviral diseases is increasing in the EU/EEA, as also reported by the ECDC [12].

To prevent DENV from spreading in temperate areas, several public health measures must be implemented promptly. It is crucial to timely notify cases, stratify the risk, prioritise target populations and interventions, and put in place a proactive surveillance system as delayed diagnosis may favour uncontrolled viral circulation. Unfortunately, asymptomatic presentations are common for dengue, therefore even effective surveillance programmes may not detect all imported vector-borne viraemic infections, which may constitute a source of autochthonous transmission [13].

The concomitant occurrence of distinct transmission events in our region sustained by three different DENV serotypes, and the occurrence of an unrelated outbreak in the north of Italy, indicate a need to reduce the risk of transmission by controlling vector populations. Reactive adulticide disinfestation alone could not prevent possible spread, while larval control remains the most cost-effective measure. But all vector control activities have a limited benefit if the general population is not encouraged to limit mosquito breeding sites and avoid human–vector contacts. Therefore, preventive strategies should strengthen communication to the general public and healthcare professionals. Moreover, a strong involvement of both private and public entities in contemporary actions aimed at controlling vector populations is required. A One Health approach is vital to prevent the emergence and spread of dengue (and other vector-borne diseases) to new temperate areas [14].

## Conclusion

Further studies, in Italy and European countries with autochthonous transmission events, are needed to better understand the magnitude of transmission in the general population (i.e. seroprevalence studies) and to better define the dynamics of transmission in big metropolitan areas where *Ae. albopictus* is the main vector (i.e. household transmission studies, vector competence studies, risk modelling, etc). This information will better guide public health actions and policies to better prevent and respond to new events.
